# Polyamine Synthesis Effects Capsule Expression by Reduction of Precursors in *Streptococcus pneumoniae*

**DOI:** 10.3389/fmicb.2019.01996

**Published:** 2019-08-29

**Authors:** Moses B. Ayoola, Leslie A. Shack, Mary F. Nakamya, Justin A. Thornton, Edwin Swiatlo, Bindu Nanduri

**Affiliations:** ^1^Department of Basic Sciences, College of Veterinary Medicine, Mississippi State University, Starkville, MS, United States; ^2^Department of Biological Sciences, Mississippi State University, Starkville, MS, United States; ^3^Section of Infectious Diseases, Southeast Louisiana Veterans Health Care System, New Orleans, LA, United States; ^4^Institute for Genomics, Biocomputing and Biotechnology, Mississippi State University, Starkville, MS, United States

**Keywords:** *Streptococcus pneumoniae*, polyamines, capsule, Leloir pathway, glycolysis, peptidoglycan, pentose phosphate pathway

## Abstract

*Streptococcus pneumoniae* (pneumococcus, Spn) colonizes the human nasopharynx asymptomatically but can cause infections such as otitis media, and invasive pneumococcal disease such as community-acquired pneumonia, meningitis, and sepsis. Although the success of Spn as a pathogen can be attributed to its ability to synthesize and regulate capsular polysaccharide (CPS) for survival in the host, the mechanisms of CPS regulation are not well-described. Recent studies from our lab demonstrate that deletion of a putative polyamine biosynthesis gene (Δ*cadA*) in Spn TIGR4 results in the loss of the capsule. In this study, we characterized the transcriptome and metabolome of Δ*cadA* and identified specific mechanisms that could explain the regulatory role of polyamines in pneumococcal CPS biosynthesis. Our data indicate that impaired polyamine synthesis impacts galactose to glucose interconversion via the Leloir pathway which limits the availability of UDP-galactose, a precursor of serotype 4 CPS, and UDP-*N*-acetylglucosamine (UDP-GlcNAc), a nucleotide sugar precursor that is at the intersection of CPS and peptidoglycan repeat unit biosynthesis. Reduced carbon flux through glycolysis, coupled with altered fate of glycolytic intermediates further supports impaired synthesis of UDP-GlcNAc. A significant increase in the expression of transketolases indicates a potential shift in carbon flow toward the pentose phosphate pathway (PPP). Higher PPP activity could constitute oxidative stress responses in Δ*cad*A which warrants further investigation. The results from this study clearly demonstrate the potential of polyamine synthesis, targeted for cancer therapy in human medicine, for the development of novel prophylactic and therapeutic strategies for treating bacterial infections.

## Introduction

*Streptococcus pneumoniae* (pneumococcus) is a Gram-positive bacterial pathogen that can cause infections, such as sinusitis, otitis media, meningitis, septicemia, and most commonly, community-acquired pneumonia (Musher, 1992; Greenwood, 1999). Phase variation enables Spn to modulate CPS expression during nasopharyngeal colonization or invasion of sterile sites (Manso et al., 2014; Li J. et al., 2016). The pneumococcal capsule interferes with opsonization and phagocytosis, and is also the basis for classification of nearly 100 identified serotypes (Geno et al., 2015). Capsule biosynthesis in pneumococcus generally follows the canonical Wzy-dependent pathway, which is common to the majority of the serotypes including serotype 4 (TIGR4) which is the focus of this study. In Wzy-dependent mechanism, CPS repeat units are built on the inner side of the cytoplasmic membrane, transported to the outer side by a flippase, further polymerized by a Wzy polymerase, and covalently linked to the cell wall PG (Eberhardt et al., 2012). Although pneumococcal capsules are well characterized, the description of molecular mechanisms that regulate CPS biosynthesis is limited.

The capsule biosynthesis locus is located between the *dexB* and *aliA* genes in the pneumococcal genome and constitutes a single operon with genes *cpsA-O*, depending on serotype. Transcriptional regulation of CPS expression is reported to involve the first four genes *cpsA*–*cpsD* of the *cps* locus (Morona et al., 2004; Hanson et al., 2011; Ghosh et al., 2018), the promoter sequence upstream of *cpsA* (Wen et al., 2015), and competence protein (ComE) (Zheng et al., 2017). In addition to transcriptional regulation, recent studies identified effects on central metabolism that impact CPS. Uracil deprivation and impaired pyruvate oxidase, an important enzyme in the production of acetyl-CoA, have both been shown to result in reduced CPS (Echlin et al., 2016; Carvalho et al., 2018). An arginine/ornithine antiporter, *arcD*, has also been reported to regulate CPS biosynthesis by unknown mechanisms (Gupta et al., 2013). A comprehensive description of the regulatory framework for the expression of this critical virulence factor in pneumococci is still a work in progress.

Polyamines such as spermidine, putrescine, and cadaverine are ubiquitous, polycationic, aliphatic hydrocarbons with amino groups that regulate a number of cellular processes (Bae et al., 2018). We have shown that isogenic deletion of *cadA*, a gene that encodes a putative lysine decarboxylase that catalyzes the conversion of lysine to cadaverine resulted in an attenuated phenotype in murine models of pneumococcal colonization, pneumonia, and sepsis (Shah et al., 2011). Characterization of *S. pneumoniae* TIGR4 Δ*cadA* showed a loss of the capsule (Nakamya et al., 2018), which in part, could be due to transcriptional downregulation of *cps4A*, the first gene in the *cps* operon. Expression proteomics analysis of Δ*cadA* indicated a shift in central metabolism that could limit the availability of precursors for CPS synthesis. However, the limited proteome coverage failed to identify specific molecular mechanisms of CPS regulation.

To identify pneumococcal pathways responsive to polyamines that regulate CPS synthesis, we compared the transcriptomic and metabolomic profiles of *S. pneumoniae* TIGR4 and Δ*cadA* strains. Our results show that the ability to convert UDP-Glu to UDP-Gal through Leloir pathway was reduced in Δ*cadA*. Observed changes in the expression of genes in the amino and nucleotide sugar metabolism are expected to result in reduced intracellular levels of UDP-GlcNAc, a precursor for three *N*-acetylated sugars in the serotype 4 capsule repeat unit. Impaired Leloir pathway and UDP-GlcNAc synthesis will limit the availability of CPS precursors. Changes in the metabolism in Δ*cadA*, such as reduced glycolytic activity, altered UDP-GlcNAc metabolism and lysine synthesis could impact the assembly of PG and ultimately the cell wall which provides attachment for the capsule. In summary, using RNA-Seq and metabolomics, we identified specific mechanisms in polyamine synthesis deficient pneumococci, which could limit the availability of precursors for CPS and PG synthesis. This study is the first report on the impact of altered polyamine metabolism, often reported as a target for cancer therapy in human medicine, on bacterial pathogenesis. The results from this study clearly demonstrate the potential of targeting polyamine synthesis in bacteria for the development novel prophylactic and therapeutic strategies.

## Materials and Methods

### Bacterial Strains and Growth Conditions

*Streptococcus pneumoniae* serotype 4 strain TIGR4 and Δ*cadA* were used in this study. All strains were grown in Todd-Hewitt broth supplemented with 0.5% yeast extract (THY) or on 5% sheep blood agar plates (BAP) in 5% CO_2_. Generation of Δ*cadA* and initial characterization of CPS are described elsewhere (Nakamya et al., 2018). All strains were grown to mid-log phase (O.D_600 nm_ 0.4) and cells were pelleted, for extraction of total RNA and metabolites. Colony forming units (CFUs) were enumerated to ensure comparable number of working cells.

### RNA Sequencing

Total RNA was isolated and purified from mid-log phase cultures of TIGR4 and Δ*cadA* grown in THY (*n* = 3) using the RNeasy Midi Kit and QIAcube (Qiagen, Valencia, CA, United States). The quality was analyzed by an Agilent 2100 Bioanalyzer (Agilent Technologies, Santa Clara, CA, United States). Libraries for RNA-Seq were prepared with the KAPA RNA Hyper Kit with RiboErase (KAPA Biosystem, Wilmington, MA, United States) with 5 μg RNA as input. The workflow consists of rRNA removal, cDNA generation, end repair to generate blunt ends, A-tailing, adaptor ligation, and PCR amplification. Different adaptors were used for multiplexing samples in one sequencing run. Library concentrations and quality were measured using Qubit ds DNA HS Assay Kit (Life Technologies, Carlsbad, CA, United States) and Agilent Tapestation (Agilent Technologies, Santa Clara, CA, United States). Sequencing was performed on an Illumina Hiseq 3000 for a single read 50 run. Data quality check was done on Illumina SAV. De-multiplexing was performed with Illumina Bcl2fastq2 v 2.17. Analysis to remove failed reads, mapping of the short sequence reads to Spn TIGR4 reference genome, and identification of differentially expressed genes were performed with RNA-Seq tool of CLC Genomic Workbench 11.0.1 (Qiagen, Valencia, CA, United States). Briefly, single end reads of both wild type and mutant were mapped to the *S. pneumoniae* TIGR4 genome using CLC proprietary read mapper, counted with EM estimation algorithm, and differentially expressed genes were identified based on the fold change generated by the edgeR algorithm. Fold change with false discovery rate (FDR) ≤ 0.05 was accepted to be significant. RNA-Seq raw data and metadata reported in this study are available at NCBI GEO with the accession number GSE130511. The differentially expressed genes were analyzed by integrating multiple bioinformatics resources such as KEGG (Kanehisa et al., 2012), UniProt (The UniProt Consortium, 2018), STRING (Szklarczyk et al., 2019), DAVID (Huang da et al., 2009), and EcoCyc (Keseler et al., 2017) to infer biological functions altered in Spn serotype 4 harboring a gene deletion in a putative lysine decarboxylase.

### UPLC–HRMS Untargeted Metabolomics Analysis

*Streptococcus pneumoniae* TIGR4 and Δ*cadA* strains were cultured in THY (mid-log phase, ∼10^9^ CFU, *n* = 5) and transferred onto a 0.2 μm Whatman polycarbonate membrane by vacuum filtration. The membranes were flash-frozen in liquid nitrogen and stored at −80°C until further use. Metabolites from bacteria collected on membrane were extracted by placing the membranes into petri dishes with 1.3 ml of extraction solvent pre-chilled to 4°C (40:40:20 HPLC grade methanol, acetonitrile, and water with 0.1% formic acid). The extraction was allowed to proceed for 15 min at −20°C. The membranes were flipped over and rinsed with the extraction solvent. The extraction solvent was transferred to 2.0 ml centrifuge tubes and an additional 300 μl of extraction solvent was added to each membrane. The membranes were compressed and rinsed to extract the remaining cells and the extraction solvent was transferred to a centrifuge tube. The centrifuge tubes with extraction solution were centrifuged for 5 min (16,100 × *g*) at 4°C to remove cellular debris and the supernatant was transferred to new 2.0 ml tubes. The residual cells were resuspended with 100 μl of extraction solvent. Extraction was allowed to proceed for another 15 min at −20°C, centrifuged for 5 min (16,100 × *g*) at 4°C to remove any remaining cells and the supernatant was collected. Tubes containing ∼1.7 ml of the total supernatant were dried under a stream of N_2_ and solid residue was resuspended in 300 μl of sterile water and transferred to 300 μl autosampler vials. Samples were immediately placed in autosampler trays for mass spectrometric analysis.

Samples placed in an autosampler tray were kept at 4°C. A 10 μl aliquot was injected through a Synergi 2.5-micron reverse-phase Hydro-RP 100, 100 × 2.00 mm LC column (Phenomenex, Torrance, CA, United States) kept at 25°C. The eluent was introduced into the MS via an electrospray ionization source conjoined to an Exactive^TM^ Plus Orbitrap Mass Spectrometer (Thermo Scientific, Waltham, MA, United States) through a 0.1 mm internal diameter fused silica capillary tube. The mass spectrometer was run in full scan mode with negative ionization mode with a window from 85 to 1000 *m*/*z* with a method adapted from Lu et al. (2010). The samples were run with a spray voltage of 3 kV. The nitrogen sheath gas was set to a flow rate of 10 psi with a capillary temperature of 320°C. Automatic gain control (AGC) target was set to 3e6. The samples were analyzed with a resolution of 140,000 and a scan window of 85–800 *m*/*z* from 0 to 9 min and 110–1000 *m*/*z* from 9 to 25 min. Solvent A consisted of 97:3 water:methanol, 10 mM tributylamine, and 15 mM acetic acid. Solvent B was methanol. The gradient from 0 to 5 min is 0% B, from 5 to 13 min is 20% B, from 13 to 15.5 min is 55% B, from 15.5 to 19 min is 95% B, and from 19 to 25 min is 0% B with a flow rate of 200 μl/min.

Files generated by Xcalibur (RAW) were converted to the open-source mzML format (Martens et al., 2011) via the open-source msconvert software as part of the ProteoWizard package (Chambers et al., 2012). Maven (mzroll) software, Princeton University (Melamud et al., 2010; Clasquin et al., 2012) was used to automatically correct the total ion chromatograms based on the retention times for each sample (Clasquin et al., 2002; Melamud et al., 2010). Metabolites were manually identified and integrated using known masses (±5 ppm mass tolerance) and retention times (Δ ≤ 1.5 min). Unknown peaks were automatically selected via Maven’s automated peak detection algorithms. We used a database of 275 metabolites verified using exact *m*/*z* and known retention times, expanded from the original database of Lu et al. (2010) as additional standards become available. The statistical analysis on metabolite peak intensity post CFU normalization was done by MetaboAnalyst 4.0 (Chong et al., 2018). We used quantile normalization that has been reported to be highly efficient in normalizing metabolite variations from mass spectrometry to normalize our data (Lee et al., 2012). Significant differences in metabolite peak intensity between Δ*cadA* and TIGR4 were identified by a *T*-test at an adjusted FDR of 0.05 (Li et al., 2017). Sparse partial least squares-discriminant analysis (sPLS-DA) was used for the statistical data presentation. We used KEGG (Kanehisa et al., 2012) and EcoCyc (Keseler et al., 2017) to infer metabolic pathways in *S. pneumoniae* TIGR4 represented by our untargeted metabolomics data.

### Measurement of Pyruvic Acid

To measure the glycolytic pathway activity, we estimated the concentration of secreted pyruvate, the end product of glycolysis, using the Pyruvic Acid Assay Kit (Megazyme, Bray, Ireland) following the manufacturer’s protocols. Briefly, mid-log phase cultures of TIGR4 and Δ*cadA* were pelleted at 6000 × *g* for 5 min. Ten microliters of supernatant was added to a mixture of 240 μl distilled water, 20 μl of assay buffer, and 10 μl of assay solution 2 containing NADH. Ten microliters of provided assay standard solution and 10 μl of distilled water were used in place of supernatant for the standard mix and blank mix, respectively. The mixtures were incubated for 2 min at room temperature (25°C). Addition of 2 μl of solution 3 containing D-LDH was used in initiating the pyruvate quantification reaction. After a reaction time of 3 min, the absorbance was read at 340 nm. Excreted pyruvate was estimated using the formula below:

Pyruvate(μg/mL)=(ΔA-sampleb)/m,

where Δ*A*_sample_ is the absorbance of the sample, b is the y-intercept, and m is the slope (from the linear equation generated from the assay).

Pyruvate(g/l)=(ΔA/sampleΔA)standard×g/lstandard,

where Δ*A* is the absorbance measurement.

The amount of pyruvate produced by *S. pneumoniae* TIGR4 and Δ*cadA* strains was normalized using estimated pneumococcal total protein according to the BCA method (Smith et al., 1985), and the result presented as pyruvate (ng)/protein (μg).

### Estimation of Surface Exposed Phosphocholine

Fluorescence-activated cell sorting (FACS) comparison of surface exposed phosphocholine (PC) levels between the wild type and Δ*cadA* strains was performed (Shainheit et al., 2014) to validate our reported loss of CPS and higher expression of choline binding proteins (CBPs) in Δ*cadA* (Nakamya et al., 2018). Briefly, 300 μl of mid-exponential-growth-phase bacteria was pelleted and washed in 1× PBS. Pellets were resuspended in 100 μl of unconjugated IgA, Kappa from murine myeloma anti- PC (Sigma–Aldrich, St. Louis, MO, United States) at 1:100 in 1× PBS and incubated on ice for 30 min. The binding reaction was stopped with 500 μl of 1× PBS and centrifuged at 4,000 × *g* for 5 min. Pellets were resuspended in 100 μl of phycoerythrin (PE)-conjugated rat anti-mouse IgA secondary antibody (ThermoFisher Scientific, Waltham, MA, United States) at 1:100 in PBS and incubated at 4°C, in the dark, for 30 min. Staining reactions were stopped with 500 μl PBS, and products were pelleted and resuspended in 300 μl of 2% paraformaldehyde (PFA). Samples were collected (10,000 events), analyzed, and plotted using an Attune Acoustic Focusing Cytometer (Life Technology, Foster City, CA, United States).

## Results

### Effect of *cadA* Deletion on Pneumococcal Gene Expression

RNA-Seq-based comparative transcriptome analysis of *S. pneumoniae* TIGR4 and Δ*cadA* identified significant changes in the expression of 432 genes, of which 179 were downregulated and 253 were upregulated in Δ*cadA*. Biological functions and pathways represented by genes responsive to the impaired putative lysine decarboxylase gene are discussed in the following sections and shown in Tables 1–4.

**TABLE 1:** Differentially expressed genes in Δ*cadA* from capsule biosynthesis pathways.

	**Locus**		**Fold**
**Gene**	**tag**	**Description**	**change**
*Aga*	SP_1898	Alpha-galactosidase	–2.7
*galK*	SP_1853	Galactokinase	–5.0
*galT2*	SP_1852	Galactose-1-phosphate uridylyltransferase 2	–5.9
*nanB*	SP_1687	Sialidase B	–3.7
*SP_2167*	SP_2167	Putative L-fuculose kinase fucK	4.1
*fucA*	SP_2166	L-Fuculose phosphate aldolase	3.7
*SP_2168*	SP_2168	Putative fucose operon repressor	1.9
*SP_0321*	SP_0321	PTS system, IIA component	–2.4
*SP_0323*	SP_0323	PTS system, IIB component	–3.7
*SP_0324*	SP_0324	PTS system, IIC component	–3.7
*SP_0325*	SP_0325	PTS system, IID component	–3.5
*glgB*	SP_1121	1,4-Alpha-glucan branching enzyme	2.1
*glgC*	SP_1122	Glucose-1-phosphate adenylyltransferase	1.7
*glgD*	SP_1123	Glycogen biosynthesis protein	1.6
*glgA*	SP_1124	Glycogen synthase	1.7
*malP*	SP_2106	Maltodextrin phosphorylase	–2.1
*malQ*	SP_2107	4-Alpha-glucanotransferase	–2.8
*exp5*	SP_0758	PTS system glucose-specific EIICBA component	–1.6
*SP_1795*	SP_1795	Putative sucrose-6-phosphate hydrolase	–3.0
*glmS*	SP_0266	Glutamine-fructose-6-phosphate aminotransferase	–1.6
*nagA*	SP_2056	*N*-acetylglucosamine-6-phosphate deacetylase	2.9
*nagB*	SP_1415	Glucosamine-6-phosphate deaminase	3.9
*pyrR*	SP_1278	Bifunctional protein PyrR	–1.9
*SP_1163*	SP_1163	Putative acetoin dehydrogenase, beta subunit	–1.5
*SP_1164*	SP_1164	Putative acetoin dehydrogenase, alpha subunit	–1.4

**TABLE 2:** Differentially expressed genes in Δ*cadA* involved in PG and choline binding protein synthesis, and carbon utilization pathways.

	**Locus**		**Fold**
**Gene**	**tag**	**Description**	**change**
Peptidoglycan biosynthesis			
*glnH*	SP_0609	Amino acid ABC transporter, amino acid-binding protein	–1.6
*glnP*	SP_0607	Amino acid ABC transporter, permease protein	–1.5
*glnQ*	SP_0610	Amino acid ABC transporter, ATP-binding protein	–1.7
*Asd*	SP_1013	Aspartate-semialdehyde dehydrogenase	–5.4
*dapA*	SP_1014	4-hydroxy-tetrahydrodipicolinate synthase	–4.8
*lys9*	SP_0919	Saccharopine dehydrogenase	–26.4
*SP_1994*	SP_1994	Aminotransferase, class I	–1.4
*murI*	SP_1881	Glutamate racemase	–1.7
Choline binding protein synthesis			
*pspA*	SP_0117	Pneumococcal surface protein A	2.3
*cbpA*	SP_2190	Choline binding protein A	1.8
*cbpI*	SP_0069	Choline binding protein I	1.5
*pcpA*	SP_2136	Choline binding protein PcpA	2.3
Carbon utilization pathways			
*SP_0877*	SP_0877	PTS system, fructose-specific IIABC components	2.6
*SP_0645*	SP_0645	Putative PTS system, IIA component	3.1
*SP_0646*	SP_0646	Putative PTS system, IIB component	2.6
*SP_0647*	SP_0647	Putative PTS system, IIC component	2.6
*lacD*	SP_1190	Tagatose 1,6-diphosphate aldolase	1.9
*lacC*	SP_1191	Tagatose-6-phosphate kinase	1.9
*lacB*	SP_1192	Galactose-6-phosphate isomerase subunit	1.7
*lacA*	SP_1193	Galactose-6-phosphate isomerase subunit	1.8
*tktC*	SP_2127	Transketolase, C-terminal subunit	97.9
*tktN*	SP_2128	Transketolase, N-terminal subunit	94.1
*SP_2129*	SP_2129	Putative PTS system, IIC component	81.5
*SP_2130*	SP_2130	Putative PTS system, IIB component	68.7

**TABLE 3:** Differentially expressed genes of known virulence factors, stress response, and polyamine biosynthesis in Δ*cadA*.

	**Locus**		**Fold**
**Gene**	**tag**	**Description**	**change**
Virulence			
*livH*	SP_0750	Branched-chain amino acid ABC transporter, permease protein	–1.5
*livM*	SP_0751	Branched-chain amino acid ABC transporter, permease protein	–1.6
*livG*	SP_0752	Branched-chain amino acid ABC transporter, ATP-binding protein	–1.7
*livF*	SP_0753	Branched-chain amino acid ABC transporter, ATP-binding protein	–1.6
*metQ*	SP_0149	Lipoprotein	–1.7
Stress response			
*SP_1883*	SP_1883	Putative dextran glucosidase	–28.1
*SP_1884*	SP_1884	Trehalose PTS system, IIABC components	–26.2
*psaB*	SP_1648	Manganese ABC transporter, ATP-binding protein	2.7
*psaC*	SP_1649	Putative manganese ABC transporter, permease protein	2.7
*psaA*	SP_1650	Manganese ABC transporter substrate-binding lipoprotein	2.7
Polyamine biosynthesis			
*aguA*	SP_0921	Putative agmatine deiminase	–25.7
*aguB*	SP_0922	Carbon–nitrogen hydrolase family protein	–19.7
*nspC*	SP_0920	Carboxynorspermidine decarboxylase	–27.5
*speE*	SP_0918	Polyamine aminopropyltransferase	–41.5

**TABLE 4:** Differentially expressed two component system genes in Δ*cadA*.

		**Fold**		
**Gene**	**Locus**	**change**	**TCS**	**Role**
*comDE*	SP_2235/6	−1.7/−2.0	TCS12	Competence and virulence
*ciaRH*	SP_0798/9	−1.7/−1.8	TCS05	Competence, virulence, and antibiotics
*SP_2000/1*	SP_2000/1	−1.5/−1.7	TCS11	No known impact on virulence
*SP_0661*	SP_0661	1.3	TCS09	Transition from lung to blood
*SP_2193*	SP_2193	1.4	TCS06	Transcriptional regulator of CbpA
*SP_1632/3*	SP_1632/3	1.6/1.4	TCS01	No known impact on virulence

#### Impaired Polyamine Synthesis Reduces the Expression of Genes That Regulate Intracellular Concentrations of Precursors for Capsular Polysaccharide Synthesis

*Streptococcus pneumoniae* TIGR4 genes responsive to impaired polyamine synthesis have functions that are directly involved in the metabolism and transport of CPS precursors (Table 1). The acetylated sugars in the repeat unit of serotype 4 polysaccharide structure are synthesized from the following nucleotide sugar precursors: UDP-Gal, UDP-ManNAc, UDP-FucNAc, and UDP-GalNAc (Jones et al., 1991; Geno et al., 2015). Our results show that the expression of genes involved in the synthesis and availability of sugar precursors in the repeat unit is significantly altered in Δ*cadA*. Expression of alpha-galactosidase (*aga*), which cleaves galactose from raffinose and melibiose (Rosenow et al., 1999), is downregulated (Table 1). Galactokinase (*galK*) and galactose-1-phosphate uridylyltransferase 2 (*galT2*), the genes encoding key enzymes of Leloir pathway, a predominant route for galactose catabolism and generation of UDP-Gal, are significantly downregulated. We observed downregulation of sialidase B (*nanB*), an enzyme that catalyzes the cleavage of terminal sialic acid from host glycoconjugates to ultimately generate UDP-ManNAc. Genes that encode fuculose kinase and fuculose phosphate aldolase are upregulated in Δ*cadA*. These enzymes are responsible for the degradation of fucose, a sugar component of CPS repeat unit structure. Glycerone 3-phosphate produced from fucose degradation can be converted by a triosephosphate isomerase directly to G3P, an intermediate of glycolysis and PPP (Higgins et al., 2014). Our data also show the upregulation of a putative fucose operon repressor (*SP_2168*) that could inhibit fucose utilization. All genes encoding the phosphotransferase system (PTS) IIA-D components specific for the import of *N*-acetylgalactosamine, a sugar that is part of the CPS repeat unit, are downregulated.

**FIGURE 1:**
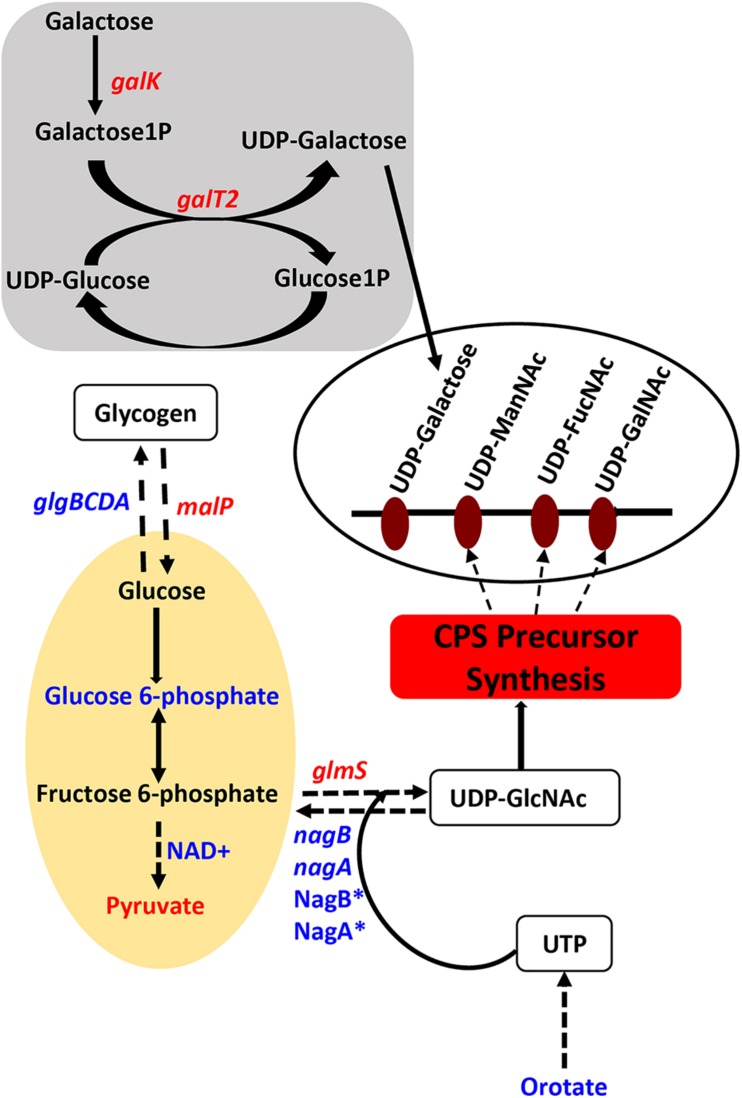
Impaired polyamine synthesis in *S. pneumoniae* impacts the availability of precursors for capsule (CPS) synthesis. Characterization of a putative lysine decarboxylase deletion strain of *S. pneumoniae* TIGR4 by RNA-Seq and metabolomics identified changes (blue represents increase and red represents decrease) in the genes (italicized) and metabolites in the deletion strain relative to the wild type that could explain the previously reported loss of the capsule. The deletion strain is impaired in the interconversion of UDP-Glu and UDP-Gal via the Leloir pathway (gray square) due to the reduced expression of *galk* and *galT2* genes, that can reduce the availability of UDP-Gal precursor of the repeat unit of the serotype 4 capsule (open white oval). Carbon flux through glycolysis (yellow oval) is reduced in Δ*cadA* confirmed by the accumulation of glucose 6-phosphate and NAD^+^, intermediates of the pathway and reduced levels of the end product pyruvate. Enzymatic reactions are shown as black arrows and multi-step reactions are represented by a broken arrow. Upregulation of *N*-acetylglucosamine-6- phosphate deacetylase (NagA^∗^) and GlcN6P deaminase (NagB^∗^) that could degrade intermediates of UDP-GlcNAc, at the protein level, was earlier reported and was observed at the RNA level in this study. Reduced synthesis of UDP-GlcNAc due to reduced expression of *glmS* and increased degradation due to higher expression of *nagA* and *nagB* will limit the availability of this nucleotide sugar substrate for epimerases that synthesize all three acetylated UDP-sugars of the repeat unit. Accumulation of orotate indicates impaired ability to synthesize UTP, a precursor for UDP-GlcNAc synthesis. The net effect of these changes in metabolism in Δ*cadA* results in the reduced availability of precursors for CPS synthesis (red rectangle).

Genes that encode enzymes that ultimately control the intracellular concentrations of UDP-GlcNAc are differentially expressed in Δ*cadA* (Table 1 and Figure 1). Expression of a glycogen synthesis operon (*glgBCDA*) which converts glucose 1-phosphate to glycogen was upregulated, while the expression of *malP*, which reverses this anabolic process was downregulated. This in turn will limit the availability of glucose for the biosynthesis of UDP-GlcNAc, an important substrate for epimerases that catalyze the biosynthesis of the sugars in the repeat unit of CPS. UDP-GlcNAc is converted to UDP-ManNAc and UDP-GalNAc by UDP-*N*-acetylglucosamine-2-epimerase and UDP-Glu 4-epimerase, respectively (Kay et al., 2016; Chen et al., 2018). UDP-GlcNAc can also be converted to UDP-FucNAc in a multi-step reaction involving SP_0358-60 protein (Kay et al., 2016). Genes that are involved in the degradation of maltose to glucose (*malQ*), import of glucose into the cell (*exp5*), and the generation of glucose 6-phosphate and fructose 6-phosphate from sucrose (*SP_1795*), are downregulated in Δ*cadA*. Glucose, glucose 6-phosphate, and fructose 6-phosphate, the first three intermediates of glycolysis, are precursors for UDP-GlcNAc. We also observed downregulation of *glmS* which encodes a glutamine – fructose-6-phosphate aminotransferase that converts fructose 6-phosphate to GlcN6P in UDP-GlcNAc biosynthesis, and upregulation of *nagA* and *nagB*, also at the protein level (Nakamya et al., 2018), that convert UDP-GlcNAc intermediates to fructose 6-phosphate. The net effect of gene expression changes described could be reduced concentrations of the saccharides that constitute the repeat unit that ultimately results in reduced CPS in Δ*cadA* (Figure 1).

We further identified downregulation of *pyrR*, a gene that encodes a bifunctional enzyme that regulates the interconversion of uracil and uridine monophosphate (UMP). Uracil is known to be a metabolic regulator of CPS biosynthesis in pneumococcus (Carvalho et al., 2018). In addition, UMP is the precursor for UTP that is essential for the activation of saccharides in the repeat unit as it provides UDP required to form UDP-sugars. There was also lower expression of the pyruvate dehydrogenase complex (*SP_1163* and *SP_1164*) responsible for the production of acetyl-CoA that is needed for the acetylation of sugars in the repeat unit of TIGR4.

#### Impaired Polyamine Synthesis Regulates the Expression of Genes Involved in the Synthesis of Peptidoglycan and Choline-Binding Proteins

**FIGURE 2:**
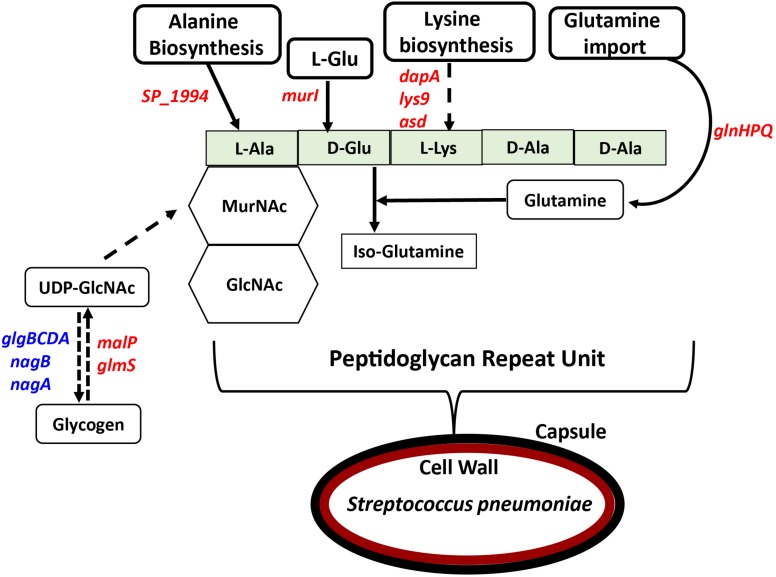
Impact of impaired polyamine synthesis on PG biosynthesis. The PG repeat unit structure in pneumococci constitutes a disaccharide (hexagon) composed of GlcNAc and MurNAc that is linked to an L-Ala, D-Glu, L-Lys, D-Ala, and D-Ala pentapeptide (light green rectangle). RNA-Seq-based comparative transcriptome analysis of Δ*cadA* impaired in polyamine synthesis and the wild type *S. pneumoniae* TIGR4 identified gene (italicized) expression changes (increase in blue and decrease in red) that could impact the PG repeat unit synthesis and cross-linking. Enzymatic reactions are shown as black arrows and multi-step reactions are represented by a broken arrow. Reduced synthesis of UDP-GlcNAc due to reduced expression of *malP* and *glmS*, and its increased breakdown due to higher expression of *nagA*, *nagB*, and the *glgBCDA* operon, will impact PG synthesis. Reduced expression of *SP_1994* that converts pyruvate to alanine, *murI* that converts L-Glu to D-Glu, and lysine biosynthesis genes (*dapA*, *lys9*, and *asd*) will limit the availability of these three amino acids in PG pentapeptide synthesis. Crosslinking of the PG repeat unit requires conversion of D-Glu to iso-glutamine using glutamine as a co-factor. Impaired glutamine import, due to reduced expression of *glnHPQ* was observed in Δ*cadA.* The overall impact of polyamine synthesis impairment is altered cell wall biosynthesis (red oval), due to specific effects on PG synthesis. The cell wall provides the point of structural attachment for the capsule (black oval) in *S. pneumoniae*.

In previous work, we reported that deletion of *cadA*, which encodes a putative lysine decarboxylase, resulted in significantly reduced expression of penicillin-binding protein 2X (Pbp2X) and choline kinase (Pck), proteins involved in PG and teichoic acid biosynthesis (Nakamya et al., 2018). In this study, we identified reduced expression of genes encoding a glutamine transporter complex: *glnH*, *glnP*, and *glnQ* in Δ*cadA* (Table 2). Glutamine is known to be directly involved in the crosslinking of PG. It serves as an important cofactor in the amidation of glutamate to iso-glutamine in the PG disaccharide–pentapeptide repeat unit, a substrate of penicillin binding protein (Zapun et al., 2013). Genes that encode proteins involved in biosynthesis of amino acids lysine (*asd*, *dapA*, and *lys9*), alanine (*SP_1994*), and D-Glu from L-glutamate (*murI*), that are components of the PG repeat unit, are all downregulated in polyamine synthesis-deficient pneumococci. Reduced expression of genes that control intracellular concentrations of UDP-GlcNAc described in the previous section could also negatively impact the PG and, ultimately, cell wall biosynthesis. UDP-GlcNAc is an essential intermediate molecule in PG biosynthesis. It is one of the disaccharide components of the PG repeat unit, and also a precursor for the biosynthesis of MurNAc, the second sugar of the repeat unit (Figure 2). These results indicate that polyamine biosynthesis regulates pneumococcal cell wall biosynthesis, a structure to which the pneumococcal capsule is attached (Larson and Yother, 2017; Figure 2).

Impaired polyamine synthesis impacts expression of genes encoding pneumococcal surface protein A, CBPs A and I, and PcpA which were upregulated in Δ*cadA*. These data suggest that deletion of *cadA* does not only mediate loss of the capsule and expose CBPs on the cell surface, but also enhance the expression of CBPs. Our earlier report indicated that there is no significant difference in the ability of Δ*cadA* to colonize the murine nasopharynx with respect to the parent strain (Shah et al., 2011). In this study, the observed upregulation of expression of CBPs could represent a response to genetic cue by pneumococcal defense machinery to enhance adherence and colonization, to overcome the expected clearance by opsonophagocytosis due to the reported loss of the capsule. Comparison of surface exposed PC using an IgA anti-PC antibody and FACS assay indicates higher signal for PC with Δ*cadA* compared to the wild-type strain (Supplementary Figure 1), validating the reported increase in the expression of CBPs at the RNA and protein levels (Nakamya et al., 2018).

#### Impaired Polyamine Synthesis Results in Increased Carbon Flux Through PPP

**FIGURE 3:**
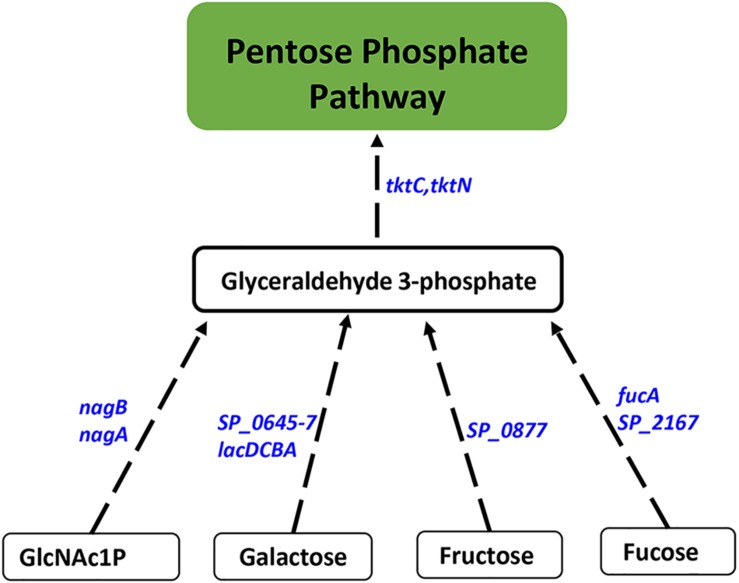
Impaired polyamine synthesis results in increased carbon flow through the PPP. RNA-Seq-based transcriptome analysis of *S. pneumoniae* TIGR4 that harbors a gene deletion in a putative lysine decarboxylase identified significant upregulation of genes (italicized, blue) in carbohydrate metabolism that indicate increased G3P, an intermediate of glycolysis, and its utilization in PPP. Enzymatic reactions are shown as black arrows and multi-step reactions are represented by a broken arrow. Increased expression of genes in metabolic pathways catalyzing the conversion of galactose (*SP_0645-7*, *lacDCBA*), fructose (*SP_0877*), fucose (*fucA*, *SP_2167*), and GlcNAc1P (*nagA*, *nagB*) to G3P with concomitant higher expression of transketolase C and N subunits (*tktC*, *tktN*) supports the increased metabolic flux through PPP.

Proteomics analysis of the polyamine synthesis deficient strain Δ*cadA* indicated a putative shift in central metabolism favoring PPP (Nakamya et al., 2018). Expression of genes responsive to *cadA* deletion include those that belong to carbon utilization pathways (Table 2). We observed upregulation of fructose-specific PTS system IIABC components, which catalyze the conversion of fructose to fructose 1-phosphate that ultimately generates G3P, an intermediate of glycolysis/PPP. This upregulation could limit the availability of fructose for the synthesis of UDP-GlcNAc. Deletion of *cadA* also resulted in the upregulation of putative PTS system IIA-C component genes and *lacDCBA*, genes involved in the import and conversion of galactose via tagatose to G3P. The net effect of these gene expression changes is expected to increase G3P levels in Δ*cadA* (Figure 3), an intermediate of glycolysis and PPP. Activation of PPP and utilization of G3P in this pathway are supported by upregulation of transketolase genes, both C- and N-terminal subunits *tktC* and *tktN*, which encode these enzymes of PPP, consistent with our earlier report of significant upregulation of transketolases at the protein level (Nakamya et al., 2018). Putative PTS system, IIBC components, which play a role in the conversion of L-ascorbate to xylulose-5-phosphate, an intermediate of PPP, are also upregulated, supporting higher PPP activity in Δ*cadA* compared to the wild-type strain, and corroborate our earlier findings of a shift in central carbon flux at the protein level (Nakamya et al., 2018).

#### Impaired Polyamine Synthesis Alters the Expression of Pneumococcal Virulence Factors and Stress Response

Expression of genes encoding branched chain amino acid transporters *livH*, *livM*, *livG*, and *livF* is downregulated in Δ*cadA* (Table 3). These transporters have been shown to contribute to pneumococcal virulence in a murine model of pneumonia and septicemia (Basavanna et al., 2009). We also observed downregulation of *metQ* that encodes methionine ABC transporter lipoprotein in Δ*cadA*. Although the exact mechanism is not known, loss of methionine transporter MetQ has been shown to lead to an attenuated phenotype in a murine model of pneumococcal pneumonia and it is reported to delay invasive disease (Saleh et al., 2014). We observed decreased expression of trehalose PTS system, IIABC components, and putative dextran glucosidase in polyamine synthesis deficient pneumococci (Table 3) which could negatively impact oxidative stress responses and the availability of glucose. Trehalose is a disaccharide containing two molecules of glucose. It is known to act as a free radical scavenger and protect yeast against oxidative stress generated by H_2_O_2_ and iron (Benaroudj et al., 2001; Jiang et al., 2018). We also observed increase in the expression of the manganese ABC transporter operon (*psaBCA*), a pneumococcal virulence factor. Evidence of role of manganese in complexes with antioxidants and other biomolecules in the detoxification of reactive oxygen species has been well documented (Culotta and Daly, 2013). Increased expression of the manganese transporter could constitute changes in oxidative stress response in Δ*cadA*.

#### Impact of Impaired Polyamine Synthesis on the Expression of Two Component Regulatory Systems

Two component regulatory systems (TCSs) are composed of a histidine kinase sensor protein and a regulatory response protein that are involved in signal transduction and adaptation of living organisms to a changing environment. A total of 13 TCSs, commonly denoted as TCS01–13, and 1 orphan regulator not coupled to a histidine kinase have been identified in pneumococcal genomes (Lange et al., 1999; Throup et al., 2000). Pneumococcal TCSs are implicated in adaptive responses to the changes in the host environment ranging from different anatomical sites, competency, environmental stress, antimicrobials, and nutrition availability that ultimately regulate pneumococcal pathogenesis (Gomez-Mejia et al., 2018). Expression of TCS01, TCS05, TCS06, TCS09, TCS11, and TCS12 is significantly altered in Δ*cadA* with respect to TIGR4 suggesting a possible role for polyamines whether upstream or downstream of TCSs (Table 4).

### Metabolic Profile of Putative Lysine Decarboxylase Deficient *S. pneumoniae*

#### Impact of *cadA* Deletion on Metabolites in Glycolysis and Capsule Biosynthesis Pathways

**FIGURE 4:**
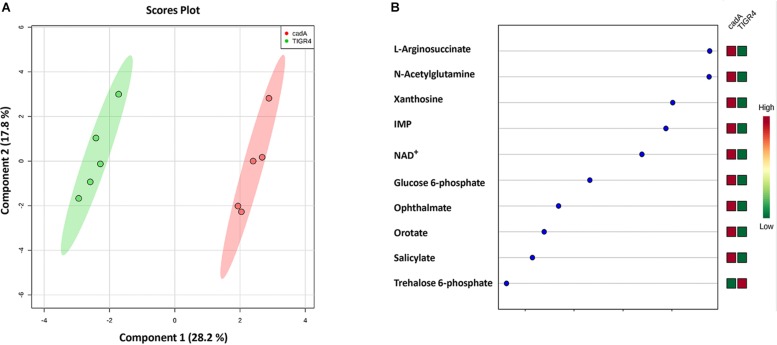
Comparison of the metabolite profiles of *S. pneumoniae* TIGR4 and Δ*cadA*. Mass spectrometry-based untargeted metabolomics analysis (*n* = 5) was performed with the wild type and polyamine synthesis deficient strain Δ*cadA.* Peak intensity values of identified metabolites were analyzed by Metaboanalyst 4.0 to identify significant changes in expression. **(A)** Clustering of samples within the sPLS-DA principal component of the wild type strain TIGR4 (green) and deletion mutant strain Δ*cadA* (pink) indicate significant metabolic differences between the two strains. **(B)** sPLS-DA loading plot of TIGR4 and Δ*cadA* with top ranked metabolites with significant differences in intracellular concentrations between the strains is shown.

**FIGURE 5:**
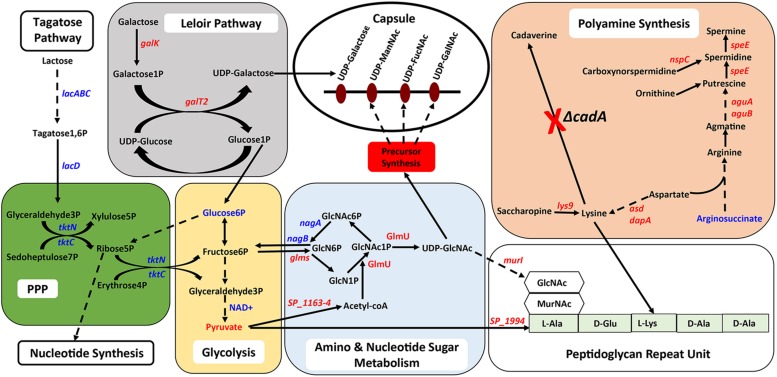
Intersection between polyamine biosynthesis, carbohydrate metabolism, and the capsule in pneumococci. Interconnected metabolic pathways in a putative lysine decarboxylase synthesis deficient strain of *S. pneumoniae* TIGR4 (Δ*cadA*) analyzed by RNA-Seq and metabolomics identified specific molecular mechanisms that could result in reduced intracellular levels of the constituents of the serotype four capsule and PG repeat structures. Enzymatic reactions are shown as black arrows and multi-step reactions are represented by a broken arrow. Changes in gene (italicized) and metabolite levels in Δ*cadA* relative to the wild-type strain are shown (red represents decrease and blue represents increase). Deletion of *cadA* results in reduced expression of genes encoding biosynthesis pathways of polyamines such as putrescine, spermidine, and spermine and precursor amino acid lysine. Accumulation of arginosuccinate, an intermediate for the biosynthesis of arginine supports the notion of overall reduction in polyamine synthesis in Δ*cadA.* Impaired polyamine synthesis renders pneumococci incapable of UDP-Glu to UDP-Gal interconversion, i.e., Leloir pathway (gray square), that could reduce the carbon flux through glycolysis (yellow rectangle). Despite increased carbon flux toward G3P, a glycolytic intermediate, through tagatose metabolism, changes in the levels of metabolites of glycolysis indicate reduced carbon flux. RNA-Seq data indicated that G3P is routed through the PPP (green square), and enhanced PPP activity will result in increased nucleotide synthesis. Changes in amino and nucleotide sugar metabolism (blue square) will result in reduced intracellular levels of UDP-GlcNAc, a precursor for three *N*-acetylated sugars in the serotype 4 capsule repeat unit (precursor nucleotide sugars shown in the open oval). Impaired Leloir pathway and UDP-GlcNAc synthesis will limit the availability of CPS precursors. Changes in metabolism in Δ*cadA*, such as reduced glycolytic activity, altered UDP-GlcNAc metabolism and lysine synthesis impacts the assembly of the PG repeat unit (open rectangle) disaccharide and pentapeptide (light green rectangle). Polymerization of PG repeat units generates the cell wall which provides attachment for the capsule.

**FIGURE 6:**
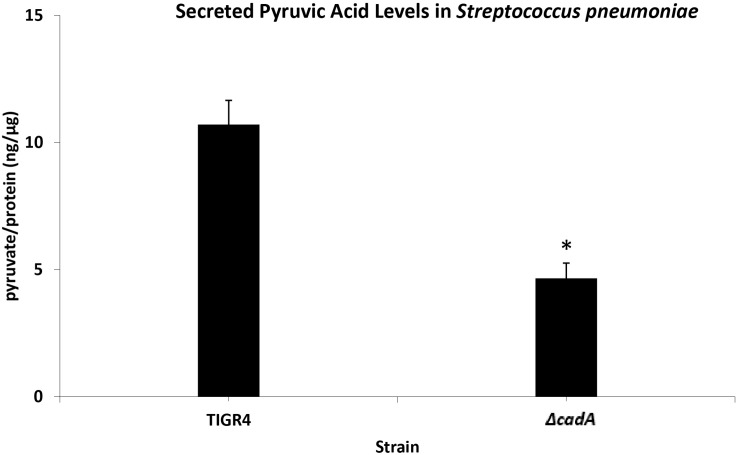
Measurement of secreted pyruvic acid from Spn TIGR4 and Δ*cadA*. Secreted pyruvate in a putative lysine decarboxylase deficient Δ*cadA* strain and Spn TIGR4 was measured using Pyruvic Acid Assay Kit (Megazyme, Bray, Ireland) according to the manufacturer’s protocol. Results show that secreted pyruvate levels in Δ*cadA* were ∼60% lower compared to the wild type strain. This data suggest that the glycolytic pathway is impaired in the polyamine synthesis deficient pneumococci. ^∗^indicate significance at *P* ≤ 0.05.

Untargeted metabolomics of TIGR4 and Δ*cadA* strains identified significant differences in the concentration of 10 metabolites (Table 5 and Figure 4). We observed accumulation of glucose 6-phosphate, an intermediate of glycolysis, indicating that flux through glycolysis is interrupted in Δ*cadA* (Figure 5). Accumulation of glucose 6-phosphate reduces the available fructose 6-phosphate that can be converted to UDP-GlcNAc, which is a precursor of repeat units in type 4 CPS (Figure 5). The levels of NAD^+^ are higher in Δ*cadA* suggesting that glucose in multi-step reactions through G3P is not being converted to pyruvate. NAD^+^ is required for the interconversion of G3P and 1,3-bisphosphoglycerate in the glycolytic pathway for generating pyruvate (Figure 5). Inhibition of glycolysis, a pathway that provides precursors for the biosynthesis of three out of four saccharides of the capsule repeat unit in Δ*cadA* could explain reduced CPS in this strain. Pyruvate is the terminal product of glycolysis and comparison of secreted pyruvate (Figure 6) showed that its concentration is significantly low in Δ*cadA* (4.66 ± 1.91 ng pyruvate/μg protein) compared to TIGR4 (10.71 ± 2.68 ng pyruvate/μg protein). Reduced levels of pyruvate in Δ*cadA* confirm reduced glycolytic activity in polyamine synthesis impaired pneumococci and support transcriptome analysis that indicates reduced availability of precursors for CPS synthesis. An additional mechanism that also has implications for CPS synthesis is the accumulation of orotate in Δ*cadA*, which suggests negative regulation of UTP synthesis. Orotate is an intermediate in the *de novo* biosynthesis of uracil, and uracil can yield UTP in a series of reversible reactions via the salvage pathway. UTP is essential to produce UDP-sugars which are substrates for the enzymes of CPS synthesis. Uracil is proposed to be a signaling molecule for CPS biosynthesis (Carvalho et al., 2018). Changes in the intracellular metabolite concentrations support the role of polyamine synthesis in modulating capsule biosynthesis.

**TABLE 5:** Significant fold changes in the intracellular levels of metabolites of Δ*cadA*.

**Metabolites**	**Fold change**
Xanthosine	4.2
Trehalose 6-phosphate	–2.5
IMP	2.5
Glucose-6-phosphate	2.2
Orotate	2.1
L-Argininosuccinate	2.0
Ophthalmate	1.8
*N*-Acetylglutamine	1.5
Salicylate	1.5
NAD^+^	2.0

#### Impact of *cadA* on Metabolites Involved in Stress Response and Polyamine Biosynthesis

Metabolomics analysis also showed that the concentration of trehalose 6-phosphate (T6P) is reduced in Δ*cadA* (Table 4). Trehalose has been shown to protect enzymes against the damaging effect of H_2_O_2_ and heat (Asad et al., 2011). It has also been reported to have a protective role against oxidative stress in pathogenic mycobacteria (Kalscheuer and Koliwer-Brandl, 2014). Consequently, the putative lysine decarboxylase gene *cadA* could be crucial for pneumococcal stress responses indirectly through the regulation of trehalose metabolism. The level of L-arginosuccinate, an intermediate of synthesis of arginine, a precursor for the biosynthesis of putrescine and spermidine, was higher in the mutant strain. This observation supports the predicted reduced synthesis of putrescine and spermidine in Δ*cadA* by reduced expression of polyamine biosynthesis genes such as *aguA*, *aguB*, *speE*, and *nspC* identified by RNA-Seq (Table 3 and Figure 5). According to the database of prokaryote operons (DOOR) (Mao et al., 2014), *aguA*, *aguB*, *speE*, and *nspC* constitute a single operon and could be under the regulation of *cadA* that is immediately upstream of this operon in the same direction of transcription.

## Discussion

Polyamines are ubiquitous polycationic molecules found in all three domains of life (Michael, 2016). They are involved in diverse biological processes ranging from protein synthesis, nucleic acid stability, cellular growth, stress responses, and pathogenicity (Gevrekci, 2017). Intracellular polyamine concentrations are tightly regulated by transport (uptake/efflux), biosynthesis, and catabolism. Polyamine-mediated modulation of host–pathogen interactions has been leveraged and successfully targeted in the design of therapeutics, primarily against protozoan pathogens (Phillips, 2018), specifically targeting the polyamine biosynthesis pathway for the treatment of human African trypanosomiasis (Yun et al., 2010). Recent advances in cancer research have implicated modulation of polyamine synthesis and transport in chemotherapy/prevention due to the requirement of polyamines in cell proliferation (Flynn and Hogarty, 2018).

Limited serotype coverage of the existing pneumococcal vaccines coupled with genomic plasticity and enhanced antimicrobial resistance mandate the development of novel treatment and prevention strategies (Berical et al., 2016; Balsells et al., 2017; Cherazard et al., 2017). Although a large number of potential protein vaccine candidates highly conserved in most pneumococcal serotypes such as PspA, PsaA, PhpA, PhtB, PcsB, and StkP are reported in the scientific literature, clinical trials are pending (Pichichero et al., 2016). Regulation of polyamine transport and synthesis has enormous potential for the discovery of novel interventions for pneumococcal colonization and invasive diseases. We and others reported that polyamine transport protein, PotD, is a potential vaccine candidate as immunization with this protein provides protection against systemic pneumococcal infection (Shah and Swiatlo, 2006) and colonization (Shah et al., 2009; Converso et al., 2017) in mice. We reported that impaired polyamine transport (Δ*potABCD*) and synthesis (Δ*speE*, Δ*cadA*) results in attenuation of virulence of *S. pneumoniae* in murine models of colonization, pneumonia, and sepsis (Shah et al., 2011). Delving further into the role of polyamine biosynthesis in pneumococcal pathogenesis, for the first time, we demonstrated that impaired polyamine synthesis results in a reduced capsule in *S. pneumoniae* and likely explains the observed attenuation *in vivo* (Nakamya et al., 2018).

In this study, we characterized the transcriptome and metabolome of pneumococcal serotype 4 strain impaired in polyamine synthesis and identified specific mechanisms which polyamines could employ, individually or collectively, in regulating capsule biosynthesis. We identified the Leloir pathway of galactose catabolism, as a crucial mechanism impaired in Δ*cadA* (Table 1 and Figure 1). Leloir pathway is the only pathway for the interconversion of galactose and glucose (Frey, 1996). Impairment of Leloir pathway limits the availability of UDP-Gal, a precursor for one of the sugars that constitute the serotype 4 capsule repeat unit. Furthermore, galactose is reported to be the most abundant sugar in the host nasopharynx (Blanchette et al., 2016), and the ability to metabolize this important monosaccharide directly correlates to pneumococcal colonization and virulence (Paixao et al., 2015). Therefore, regulation of galactose catabolism in a polyamine-dependent manner could impact pneumococcal virulence. Deletion of catabolite control protein A (Δ*ccpA*) in the serotype 2 background has been reported to alter carbon utilization pathways and capsule attachment to the cell wall when cultured in a chemically defined medium with galactose as a carbon source (Carvalho et al., 2011). Therefore, CcpA-regulated galactose metabolism is important for the attachment of pneumococcal CPS to the cell wall. In THY growth medium that provides diverse carbon sources, although RNA-Seq did not identify significant changes in the expression of *ccpA*, our earlier report identified a significant downregulation of CcpA at the protein level in Δ*cadA* (Nakamya et al., 2018), indicating that CcpA could be part of the polyamine gene regulatory network.

Additional mechanisms identified in this study that could abrogate CPS biosynthesis include impaired glycolysis that could alter amino sugar biosynthesis, and potential upregulation of PPP that could collectively result in reduced levels of UDP-GlcNAc and ultimately limit the availability of acetylated UDP-sugars for CPS synthesis. The terminal product of glycolysis, pyruvate, and pyruvate dehydrogenase complex (*SP_1163* and *SP_1164*) genes that encode enzymes for the conversion of pyruvate to acetyl-CoA are reduced and downregulated in Δ*cadA*, respectively (Table 1 and Figure 5). Reduced pyruvate will affect CPS production since pyruvate itself is a component of the serotype 4 capsule via its attachment to galactose (Kay et al., 2016) and its conversion to acetyl-CoA is essential for the acetylation of monosaccharides in the capsule repeat unit. Downregulation of pyruvate oxidase (*spxB*) and the pyruvate dehydrogenase complex resulted in reduced levels of acetyl-CoA and CPS in serotype 4 pneumococci (Echlin et al., 2016). Reduced levels of pyruvate in Δ*cadA* reported in this study cannot be attributed to transcriptional downregulation of pyruvate oxidase, as we did not identify any significant changes in the expression of *spxB* by qRT-PCR.

Gene expression changes in Δ*cadA* indicate that polyamines can negatively regulate PG synthesis in pneumococci (Table 2 and Figure 2). Since the capsule is tethered to the cell wall of pneumococcus via direct glycosidic linkage between glucose and UDP-GlcNAc in PG (Larson and Yother, 2017), changes in PG could result in the reported loss of the capsule in Δ*cadA*. Changes in PG have implications for the susceptibility of pneumococcus to antibiotics such as penicillin that target cell wall synthesis. Expression of two component system TCS05 (*ciaR*) that controls and enhances pneumococcal resistance to cell wall targeting antibiotics such as β-lactams (Guenzi et al., 1994) and its sensor *ciaH* was downregulated in Δ*cadA*. Reduced expression of *ciaRH* combined with the downregulation of PBP2x protein reported earlier (Nakamya et al., 2018) indicates that polyamines could be modulating susceptibility to antibiotics in pneumococcus. Expression of TCS01 was upregulated in Δ*cadA*, while TCS11 expression was downregulated. TCS01 and TCS11 are predicted to be involved in resistance to antibiotics (Gomez-Mejia et al., 2018). Significant decrease in the expression of TCS12 (*comDE*) (Table 4) that controls pneumococcal competence and virulence, and increased expression of TCS06, a transcriptional regulator of CbpA (Ma and Zhang, 2007), could contribute to the loss of *in vivo* fitness (Shah et al., 2011) and upregulation of CBP expression at the RNA (Table 2) and protein levels (Nakamya et al., 2018). The six TCSs identified in this study (Table 4) as differentially expressed in Δ*cadA* represent ∼46% of the 13 TCSs in pneumococcal genomes. Therefore, polyamines could be master regulators in pneumococci via their interaction with TCSs. Alternatively, polyamines could be part of the downstream regulatory network of the TCSs. Deciphering this complex polyamine/TCS regulatory network is necessary for rational vaccine/small molecule intervention strategies that target polyamine metabolism.

The *E. coli* polyamine modulon that constitutes genes whose expression is upregulated by polyamines at the level of translation is well characterized (Igarashi and Kashiwagi, 2015). Twenty genes identified as part of the polyamine modulon in *E. coli* are reported to enhance cell proliferation and viability, biofilm formation, and ability to detoxify reactive oxygen species (Igarashi and Kashiwagi, 2018). However, altered polyamine metabolism-mediated regulation of gene expression is a recently identified area of pneumococcal physiology, with implications for pneumococcal pathogenesis. With the reported link of CcpA to regulation of TCS07 and TCS12 (Carvalho et al., 2011), establishment of CcpA as pneumococcal global regulator of carbohydrate metabolism (Iyer et al., 2005), polyamine-mediated changes in expression of CcpA (Nakamya et al., 2018), and altered galactose metabolism reported in this study, *ccpA* could constitute a polyamine modulon in pneumococcus that warrants future research and validation.

This study clearly demonstrates that polyamine synthesis is required for CPS production in pneumococci. Polyamine biosynthesis genes are well conserved in all pneumococcal genomes (Shah et al., 2011) and are necessary for survival *in vivo*. Therefore, targeting this pathway is an attractive avenue for discovering novel therapeutics and constitutes an anti-virulence strategy that could offer serotype-independent coverage without impacting nasopharyngeal colonization, i.e., disarm, but not eradicate, pneumococci (Lujan and Gallego, 2016). This approach is similar to other anti-virulence strategies such as the use of epigallocatechin gallate, the most abundant constituent of green tea, to regulate pneumococcal virulence factors pneumolysin and sortase (Song et al., 2017) and Chalcone, a natural phenol to inhibit the activity of sortase in *Listeria monocytogenes* (Li H. et al., 2016). Another polyphenol, fisetin, has been reported to interfere with the binding of listeriolysin O to the cholesterol receptor and lead to eventual elimination of cytolytic activity of *L. monocytogenes* (Wang et al., 2015). Fursultiamine hydrochloride, a derivative of thiamine, has been successfully employed to transcriptionally regulate toxin and hemolysin virulence factors in *Vibrio vulnificus* (Imdad et al., 2018). Future studies focusing on uncovering the regulatory network of pneumococcal polyamine homeostasis are warranted for leveraging the therapeutic potential of this important metabolic pathway for limiting the spread of pneumococci.

## Data Availability

The datasets generated for this study can be found in the NCBI GEO, accession number GSE130511.

## Author Contributions

BN conceived, supervised, and designed the experiments. MA performed the experiments and drafted the manuscript. LS and MN performed the experiments. JT supervised the FACS analysis. BN and ES finalized the draft. All authors approved the final version of the manuscript.

## Conflict of Interest Statement

The authors declare that the research was conducted in the absence of any commercial or financial relationships that could be construed as a potential conflict of interest.

## Abbreviations

CPS: capsular polysaccharide
D-Ala: D-alanine
D-Glu: D-glutamate
G3P: glyceraldehyde 3-phosphate
GlcN1P: glucosamine 1-phosphate
GlcN6P: glucosamine 6-phosphate
GlcNAc: *N*-acetylglucosamine
GlcNAc1P: *N*-acetylglucosamine 1-phosphate
GlcNAc6P: *N*-acetylglucosamine 6-phosphate
L-Ala: L-alanine
L-Lys: L-lysine
MurNAc: *N*-acetylmuramic acid
PG: peptidoglycan
PPP: pentose phosphate pathway
Spn: *Streptococcus pneumoniae*
UDP: uridine diphosphate
UDP-FucNAc: UDP-*N*-acetylfucosamine
UDP-Gal: UDP-galactose
UDP-GalNAc: UDP-*N*-acetylgalactosamine
UDP-Glc: UDP-glucose
UDP-GlcNAc: UDP-*N*-acetylglucosamine
UDP-ManNAc: UDP-*N*-acetylmannosamine.
