# Supplementary material for: Polyamine Synthesis Effects Capsule Expression by Reduction of Precursors in Streptococcus pneumoniae
Source: Front Microbiol. 2019 Aug 29;10:1996. doi: 10.3389/fmicb.2019.01996 (PMC6727871; doi:10.3389/fmicb.2019.01996)
Supplement: Supplementary file 1 [file Image_1.pdf]

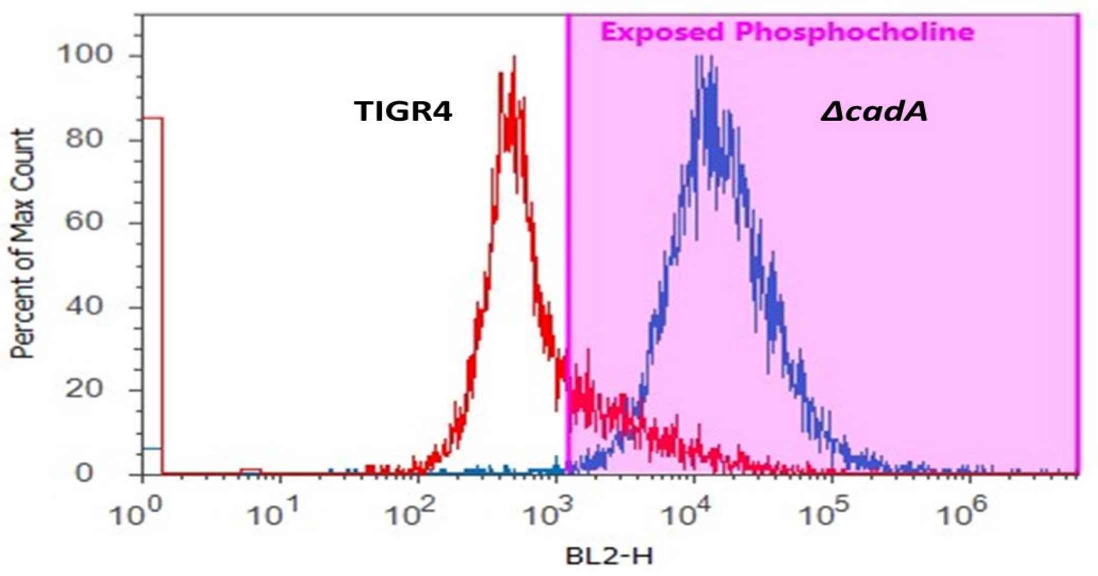

Supplementary Figure: Measurement of surface exposed phosphocholine in *S. pneumoniae* TIGR4 and *ΔcadA*. TIGR4 and *ΔcadA* strains were grown to exponential phase and surface exposed phosphocholine (PC) was measured by FACS method. PC was stained with an unconjugated Kappa murine myeloma IgA anti-phosphocholine antibody, followed by detection with a phycoerythrin-conjugated rat anti-mouse secondary antibody. Samples were fixed in 2% paraformaldehyde and read on an Attune Acoustic Focusing Cytometer. A representative histogram plot of the fluorescence intensity of murine myeloma IgA antibody binding to exposed phosphocholine on TIGR4 and *ΔcadA* is shown. The gate was set based on a negative control that was treated with secondary antibody only.
